# Effective mediation strategies for addressing social communication disorder in inclusive primary classrooms: Implications for teacher training

**DOI:** 10.4102/ajod.v15i0.1781

**Published:** 2026-01-14

**Authors:** Mashiga A. Molekoa, Moyagabo K. Malahlela

**Affiliations:** 1 Department of Inclusive Education, School of Educational Studies, University of South Africa, Pretoria, South Africa

**Keywords:** challenges, communication disorders, inclusive education, mediation strategies, primary schools, social interaction, support, teacher training

## Abstract

**Background:**

Social communication disorder (SCD) issues within inclusive primary school classrooms present significant challenges for teachers, affecting the overall teaching and learning atmosphere. Recognition of learner diversity is the cornerstone of inclusive education, whereby all learners are expected to participate equally, actively and meaningfully.

**Objectives:**

This study aimed to explore effective mediation strategies for addressing SCD in inclusive primary classrooms and how this learning disability impacts teacher professional development.

**Method:**

This study adopted a qualitative research methodology, utilising a case study design focused on four inclusive primary schools. Data were collected through in-depth, semi-structured interviews to determine individual participants’ opinions and perceptions. Thematic qualitative data analysis was used to analyse the data inductively. Twelve teachers from four inclusive primary schools were purposively selected to participate in the study.

**Results:**

The study identified several contributing factors to SCD, which can arise from various underlying causes, often related to neurological, developmental, genetic or environmental factors. Nevertheless, teachers firmly supported including learners with SCDs in inclusive classroom settings to ensure their full and equal participation alongside their peers.

**Conclusion:**

This study argues that negative teacher perceptions in schools might negatively affect the teaching and learning environment, causing long-term consequences for learners who display SCDs and their capacity to meet academic objectives.

**Contribution:**

The study may shed light on strategies to effectively curb SCDs posed by learners in inclusive primary classrooms. School Management Teams (SMTs), teachers, parents, and other relevant key players will benefit from the study’s outcomes to improve their knowledge and skills to support learners with SCDs. This study recommends integrating inclusive policy and legislation into the implementation of mediation strategies for addressing SCD in primary classrooms.

## Introduction

Inclusive education is now widely acknowledged as a key principle for providing equal learning opportunities to all learners, regardless of their abilities or disabilities (Jardinez & Natividad 2024). Today’s primary classrooms are highly diverse, with learners presenting a variety of learning needs, socio-emotional differences and behavioural issues. In South Africa, even with the variety of classroom environments, teachers are encouraged to acknowledge that every student has the potential to learn and needs support. This is emphasised in the Education White Paper No. 6 (Department of Education 2001). While inclusion fosters an equitable learning environment, managing social communication disorder (SCD) in these settings presents significant challenges for teachers. Despite the growing emphasis on inclusive education, further empirical research is needed to determine the most effective intervention strategies and how to incorporate them into teacher training programmes. Regardless of the differences in the severity of their conditions or the extent of support they need, learners with SCD remain excluded from mainstream inclusive primary classrooms (Cullen et al. 2020).

Social communication disorder is a neurodevelopmental disorder (NDD) that affects an individual’s ability to use verbal and nonverbal communication skills effectively in social interactions, which can result in social difficulties and behavioural difficulties (Topal et al. 2018). In an inclusive classroom, learners with SCD may misunderstand instructions, face difficulties in group interactions and struggle to build relationships. Individuals with SCD may find it challenging to grasp and apply the social norms of communication, making it hard for them to participate in meaningful conversations and sustain relationships (Brighter Strides ABA 2024). They may struggle to adjust their communication style based on different situations and struggle with pragmatic language. This condition can impact various forms of communication, including speaking, writing, gestures and sign language. Tee-Melegrito (2023) asserts that communication disorders impact an individual’s ability to recognise, interpret, process and understand the symbols or concepts essential for communication. These disorders can affect hearing, language and speech. The present study has explored how the teachers in inclusive mainstream primary schools of Bojanala District in North West province in South Africa have applied mediation strategies to curb the challenges related to SCD and to find out whether those strategies were effective or not. Lebopa (2018) reiterates that when teachers do not help or manage learners who are experiencing behavioural challenges [in the context of this study, the SCD], they are not following the guidelines in the South African policy on Screening, Identification, Assessment, and Support (SIAS) (Department of Education 2014). Sarkar and Kundu (2021) express concern over the apparent lack of classroom support for teachers, which poses a challenge to effectively implementing inclusive practices and mediation strategies for SCD issues (Arnaiz-Sánchez et al. 2023).

Effective education mediation strategies, which enhance learners’ awareness of their emotions, attitudes and values, can play a role in encouraging positive classroom communication (Frenzel, Daniels & Burić 2021). Effectively addressing SCD in an inclusive primary school classroom requires a combination of structured interventions, environmental modifications and teacher support strategies. A range of effective mediation strategies includes utilising visual aids, providing direct instruction on social skills, ensuring communication is simplified and clear, fostering a flexible and accommodating classroom setting, leveraging technology for communication, and emphasising teacher training along with collaborative efforts (Douglas & Gerde 2019). Research conducted by Dwarika (2019) indicates that teachers who receive training in inclusive education teaching strategies gain a deeper awareness of the broader factors influencing learners’ behaviour and critically assess the impact of their evolving teaching practices. Kahveci (2023) asserts that teachers are pivotal contributors to the education systems worldwide as they help learners develop skills, instil moral, behavioural and social values, equip them for future careers, and share academic knowledge.

This study aims to explore successful approaches to SCD mediation in inclusive primary classrooms, offering insights that could enhance teacher preparation programmes and professional development initiatives. This research is situated within the wider framework of inclusive primary education, where teachers are tasked with managing both academic instruction and the diverse social-emotional needs of their learners. The study aims to investigate and assess mediation strategies that teachers can implement to address SCD in inclusive classroom settings effectively. Specifically, this study aims to identify the common SCDs that teachers encounter in inclusive primary classrooms, evaluate teachers’ current mediation strategies and their effectiveness, and determine how well teacher training programmes prepare them to handle SCD. The study further recommends enhancing teacher training in SCD mediation within inclusive education.

### Theoretical framework

This study was conducted from the perspective of inclusive pedagogy proposed by Vygotsky (1978), with his sociocultural theory serving as the foundational framework for the investigation. In this research, sociocultural perspectives significantly shape the social interactions when a child collaborates with others and engages with different situations (Bernard 2024). As learners approach the zone of proximal development (ZPD), teachers must notice changes in their learners and embrace their role as facilitators of learning. Support from a teacher, peer or another knowledgeable adult with more expertise is crucial for fostering their growth. Vygotsky (1978) asserts that learning takes place within socio-cultural settings, where children interact and collaborate with more knowledgeable individuals through dialogue. Socio-cultural theory strongly emphasises the connection between social interactions and cognitive development. According to Vygotsky (1978), human beings can engage in advanced thinking processes stimulated by language, signs, symbols and materials through social and cultural interactions. Language development helps children to plan solutions before implementing them, manage impulsive behaviour, navigate challenging situations and develop self-control (NeuroLaunch Editorial Team 2024).

Acknowledging and valuing cultural diversity allows educational practices to be adapted to meet the diverse needs and experiences of children from various cultural backgrounds (Baheretibeb & Whitehead 2024; Montaña 2024). Moreover, promoting cultural sensitivity and inclusivity in education fosters equitable opportunities for cognitive growth and development (Jardinez & Natividad 2024). As a theoretical framework, socio-culturalism is closely linked to the concept of culture (Dai 2023). Culture encompasses the collective beliefs, values, norms, customs, behaviours, symbols and practices that define a specific group or society (Bernard 2024; Dai 2023).

The ZPD, as encapsulated by Lev Vygotsky’s socio-cultural theory, denotes the difference between a learner’s existing level of competence and the potential level they can reach with guidance and support from more knowledgeable individuals, such as teachers or peers (Main 2024). In the context of this study, the teacher, as the knowledgeable other, is responsible for guiding and supporting learners with SCDs in their classroom setups. This implies that the ZPD represents the gap between what a learner can accomplish independently and what they can achieve with support and guidance from a more skilled individual (McLeod 2024).

### Literature review

The reform plan, which involves determining how to manage learners who display SCD, primarily relies on teachers. According to a South African study by Nunan and Ntombela (2018), when teachers understand the root of the problem, they can take proactive measures and acquire the skills to mediate and prevent undesirable behaviour. The challenges come from certain innate and neurodevelopmental conditions (NDD) that result in SCD, not the learners themselves. Yoro, Fourie and Van der Merwe (2020) advocate that learners with NDD often struggle with memory weaknesses and may experience difficulties with behaviour, motor skills and speech. Teachers can support learners in dealing with SCD while encouraging the development of positive behaviour and improving social interaction within their classrooms and school communities (Lee 2022). Understanding the various types of support teachers need to be well-equipped to mediate challenges related to diversity and inclusion in the classroom is essential. By recognising the importance of positive behaviour and applying effective strategies, such as establishing clear expectations, utilising positive reinforcement, demonstrating appropriate behaviour and integrating engaging activities, teachers can greatly improve learners’ academic performance and social development (Dwarika 2019). Masedi et al. (2023) suggest that intervention strategies can be developed based on teachers’ direct role in helping learners build coping mechanisms for difficult situations in the classroom.

Social communication disorder in primary school learners can impact both academic achievement and social connections, highlighting the importance of early detection and intervention (Brinton & Fujiki 2018). Social communication disorder involves challenges in interpreting social cues such as facial expressions, body language and tone of voice; difficulties with figurative language; issues with adhering to social norms; trouble participating in cooperative play or group activities; and possible emotional and behavioural difficulties (Brighter Strides ABA 2024). Such behaviours often impede effective interactions and lead to stress in personal, educational or professional settings. According to Vain (2024), examples of SCD and accompanying challenging behaviours can harm relationships, disrupt group harmony, and introduce tension in environments that depend on collaboration and mutual respect.

Social communication disorders and the associated behavioural challenges in the inclusive primary school classroom interfere with teaching and learning, creating challenges for teachers striving to provide high-quality instruction. Chisango and Lepheana (2022) revealed that teachers face obstacles in implementing inclusive education, including inadequate teaching skills, underqualification and insufficient inclusion expertise, which hinder effective mediation strategies. Such challenges compromise learners’ academic achievement and societal contributions. Grant (2019) emphasised the need for South African teachers in mainstream schools to spearhead change for the benefit of education and diverse learners in inclusive education settings. Meanwhile, Yeboah, Charamba and Akola (2023) noted difficulties in integrating inclusive education because of a lack of active teacher development programmes and inadequate university-level preparation. Professional development workshops are therefore vital in equipping teachers to mediate behavioural issues and foster inclusive education policy and legislation.

Social communication disorder in primary school learners may result from the intricate interaction of biological and environmental influences (Mulrine & Kollia 2020). Biologically, factors such as genetic predispositions and neurological conditions play a key role in the emergence of communication disorders. For example, specific genetic mutations or a family history of speech or language impairments can elevate the risk of SCD (Mulrine & Kollia 2020). Furthermore, brain injuries, whether caused by congenital abnormalities or trauma, can impact neural pathways critical for speech and language production, leading to communication difficulties (Hangül et al. 2018). Concurrently, Cronin and Goodall (2021) attest that environmental factors significantly contribute to the development of SCD in primary school children. Socio-economic conditions, such as poverty and limited parental education, can restrict access to quality healthcare and educational resources, essential for language growth. Hoff et al. (2024) believe that children from less-educated families may have fewer opportunities to engage with diverse vocabulary and complex language patterns, affecting their communication abilities.

South Africa has aligned with global trends by shifting from the medical paradigm to the social model in addressing behavioural and learning challenges. The Salamanca Statement (UNESCO 1994) significantly propelled the global movement for inclusive education, advocating that inclusive mainstream schools are the best approach to counter discriminatory attitudes but foster harmonious communities, build inclusive societies and ensure every learner’s access to education. Wilkinson (2019) highlights that while some factors causing SCD may be beyond a child’s control, teachers’ perceptions of these causes and related situations can impact the autonomy of those involved in the classroom. This often leaves teachers feeling frustrated, powerless and uncertain about how to effectively support learners in developing socially acceptable behaviour. According to Ainscow (2020), promoting inclusion and fairness in education is less about implementing new organisational structures or practices and more about social learning processes within different contexts. To embrace the principles of inclusive education and establish friendly, supportive learning environments for all learners, teachers should possess the necessary skills and adequate knowledge to adapt their classroom activities to accommodate all learners, including those with SCD.

Mediation of challenging behaviour associated with SCD in the primary school setup involves facilitating communication, fostering understanding and helping learners recognise the impact of their actions while encouraging constructive problem-solving (Cojorn & Sonsupap 2024). Teachers, counsellors or trained mediators can conduct mediation to create a supportive environment for learners to express their thoughts, reflect on their behaviour and develop strategies for improvement.

### Objectives of the study

This study was aimed at investigating effective mediation strategies for addressing SCDs in inclusive primary classrooms of the Bojanala District, North West province, in South Africa. The study’s objective would be achieved by answering the question: How effective are the mediation strategies for addressing SCDs in inclusive primary classrooms?

## Research methods and design

The qualitative research approach was the most effective research methodology for this investigation. McMillan and Schumacher (2014) describe the qualitative approach as exploring how individuals or groups interpret social or human issues. This approach prioritises understanding the meaning of a phenomenon from the perspective of those experiencing it (Kahveci 2023). The qualitative research approach allows the researcher to gain insight into social phenomena through the perspectives and voices of the participants (Hove & Phasha 2023). The current study has employed a case study research design to examine teachers’ perceptions of the effectiveness of mediation strategies they implemented to address SCDs in their inclusive primary classrooms. Cohen, Morrison and Manion (2018) reiterate that a case study offers readers a vivid depiction of real people in real-life situations, making it easier to grasp concepts than solely relying on abstract theories or principles. In the context of this study, the case study research design has allowed the researchers to discover teachers’ insights, feelings and experiences in their endeavour to mediate and offer support to learners with SCD in their inclusive primary school settings. Cohen et al. (2018) attest that case studies typically follow the interpretive research tradition, focusing on understanding the situation from the participants’ point of view.

### Setting

The study was conducted in the Bojanala District in North West province, situated in South Africa, to the far north of Pretoria. The district is strategically located to connect four provinces: North West, Gauteng, Mpumalanga and Limpopo. The Bojanala Platinum District Municipality, classified as a Category C municipality in the North West province, shares its borders with several neighbouring regions: Waterberg District Municipality to the north, Dr Kenneth Kaunda District Municipality to the south, the City of Tshwane Metro to the east, West Rand District Municipality to the south-east and Ngaka Modiri Molema District Municipality to the west. It is one of four district municipalities in the province, encompassing five local municipalities: Kgetlengrivier, Madibeng, Moses Kotane, Moretele and Rustenburg. The area primarily consists of rural municipalities characterised by dispersed village settlements (source: Profile Analysis District Development Model 2020). The North West province is home to a diverse population of South Africans, with the Tswana people, who speak Setswana, being the predominant ethnic group. Minority communities speak Afrikaans, Sesotho and isiXhosa, while English is widely spoken across the province (Annual Performance Plan 2023–2024). To support learners with disabilities in achieving better academic outcomes, the Bojananala Department of Education ensures they have access to essential educational resources, including appropriate infrastructure. This is provided through full-service schools, inclusive education, learning and teaching support materials (LTSM), and specialised transportation (Annual Performance Plan 2023–2024).

### Study population and sampling

The study’s participants comprised two Senior Phase (Grade 7) teachers and one departmental head (DH) from the four selected inclusive primary schools, making a total of 12 (*n* = 12) participants. Purposeful sampling was used to choose information-rich participants who significantly aided in meeting the study’s informational needs to provide ideas and concepts about the effectiveness of mediation strategies in addressing SCD. Purposive sampling strategies avoid random selection and are designed to ensure that specific types of cases relevant to the research study are included in the final sample (Campbell et al. 2020). Also known as judgemental sampling, this method depends on the researcher’s discretion in choosing individuals, cases or events that are most likely to provide valuable insights for achieving the study’s objectives (Nikolopoulou 2022). The established criteria for participant selection included having a specified number of years of teaching experience, possessing a particular qualification in inclusive education, and working as a teacher or DH in the Senior Phase (Grade 7) of inclusive primary school. With the help of school principals and the school management team (SMT), identifying and selecting information-rich informants was simplified. The researchers adhered to all necessary protocols, including providing informed consent forms to participants and ensuring they understood that participation was voluntary. Additionally, the study’s aims and objectives were clearly communicated to them.

### Data collection

The researchers employed three methods for data collection: focus group discussions, in-depth interviews and observation, to enhance the effectiveness of the process and optimise the expected results (Saldana & Omasta 2018). Two focus group discussion sessions were conducted to allow participants to openly share what they feel is crucial based on their experiences, using examples from their personal stories, as promulgated by Leavy (2017). Using detailed explanations from focus group discussions and examples in the participants’ native tongue, which were subsequently translated verbatim into English, the researcher was able to gather information about the study’s main research question. The in-depth interviews, each averaging approximately 35 min in duration, served as the most important tool for gathering data in this qualitative study since they enabled active listening and aided in deciphering the significance of the experiences of the participants. According to Cohen et al. (2018:535), semi-structured forms are frequently used for in-depth interviews since they let the participants’ responses direct the discussion. This study further relied on field observations because they shed light on teachers’ real attitudes and behaviours towards learners who struggle with behaviour patterns associated with SCD in the classroom contexts. Usman and Bulut (2021) define observation as a data collection technique that entails observing individuals, objects or traits in their natural surroundings. Observations were noted during school hours when teachers were instructionally interacting with the learners in their classrooms. The researchers used field notes to record observations that the audio recorder could not capture during interviews and focus groups, particularly relevant details like participants’ emotions, psychological state and attitudes towards the questions (Modula 2022). Madiba (2023) advocates that observation is an active process involving careful attention to and documentation of objects, events and participant behaviour patterns in a study without direct interaction or communication with them.

### Data analysis

The key steps in qualitative data analysis involve organising the data into categories and identifying patterns or connections within them, primarily based on the data itself. In this study, the researchers adopted an interpretive and subjective approach to data analysis, regardless of how structured or apparent the analytical procedures seemed. After analysing the qualitative data, they aimed to identify general patterns and relationships between different data sets. Decarlo (2018) explains that the initial phase of qualitative data analysis typically involves gathering transcripts derived from focus groups or interviews. This can be achieved by taking detailed notes or, ideally, recording the sessions and later transcribing them. Furthermore, Decarlo (2018) highlights that the primary objective of qualitative data analysis is to draw insights, lessons or conclusions by refining extensive datasets into more compact and understandable segments. In this study, the researchers initially reviewed the verbatim transcripts of data collected and recorded from the focus group interviews and the in-depth interviews. Field notes were also scrutinised and categorised to gain insight into the recorded data. The data were arranged, transcribed and manually coded to inductively produce various themes and categories that shed light on the main research issues. McMillan and Schumacher (2014) explain that inductive analysis allows qualitative researchers to interpret gathered information by progressing from specific data to broader categories and patterns. Additionally, McMillan and Schumacher (2014) highlight that qualitative data analysis involves organising collected material into categories and identifying relationships among them.

### Ethical considerations

Ethical clearance was granted by the Research Ethics Review Committee of the University of South Africa in the College of Education with the reference number: Ref: 2023/11/08/33092141/45/AM. The ethical clearance certificate was obtained before the commencement of data collection. Before involvement in the study, volunteer participants were provided with detailed information about the procedures and objectives concerning the research. They were then asked to give their informed consent. It was emphasised to all participants that their participation was entirely voluntary, and they were not obligated to participate. Berg and Lune (2017) assert that it is a cornerstone of ethical research practice for subjects to knowingly consent to participate in a study without experiencing fraud, dishonesty, pressure or manipulation. Participants were assured that any information they provided would be held in strict confidence, that their identities would not be revealed in any records or reports, and that there would be no connection between the data and the participants’ real names (Modula 2022). Focus group data were anonymised by the principal researcher through the removal of personally identifiable information, such as names, contact information and any contextual hints that might disclose a participant’s identity. Participants were assigned unique codes (e.g. P-1, P-2, and so forth), with a separate linking key encrypted and stored securely in an access-controlled digital storage to enable analytical follow-up if required. The linking key will be retained only for the duration necessary to meet research or ethical obligations, and permanently deleted thereafter in accordance with institutional data protection protocols. Participants’ demographic data are illustrated in the subsequent section, under the study’s findings.

## Results

To maintain the confidentiality and privacy of the schools and participants, codes such as P-1 to P-12 (‘P’ standing for ‘Participant’) and School A to School D were used during the data analysis process. Table 1 shows the participants’ demographic data.

**TABLE 1:** Participants’ demographic data.

Participants	School codes	Gender	Years of teaching experience	Position held	Highest qualification, including knowledge of inclusive education
P-1	School A	Female	25	Gr. 7 Teacher	MEd. (Inclusive Ed.)
P-2	School A	Male	24	Dept. Head	BTech: Education Management
P-3	School A	Male	10	Gr. 7 Teacher	BEd Hons. Education
P-4	School B	Male	26	Dept. Head	BA. Ed
P-5	School B	Female	6	Gr. 7 Teacher	BEd. – Intermediate Phase
P-6	School B	Non-binary	6	Gr. 7 Teacher	BEd Hons
P-7	School C	Female	8	Gr. 7 Teacher	Postgrad. Diploma
P-8	School C	Male	23	Dept. Head	HCert, Inclusive Ed.
P-9	School C	Female	7	Gr. 7 Teacher	BEd Hons.
P-10	School D	Female	8	Gr. 7 Teacher	BEd – Intermediate Phase.
P-11	School D	Female	14	Gr. 7 Teacher	Adv. Cert. Education
P-12	School D	Non-binary	30	Dept. Head	Postgrad. Diploma in Inclusive Education

Gr., grade; Dept., department; MEd., Master of Education; BTech., Bachelor of Technology; BEd., Bachelor of Education; BA Ed., Bachelor of Arts in Education; Adv. Cert., Advanced Certificate; HCert., Higher Certificate; Ed., Education; Postgrad., Postgraduate.

Four themes have inevitably emerged from the rigorously analysed data, and have been identified as: (1) Inclusion of learners with an SCD, (2) Teacher training and continuous professional development, (3) Support provisioning for learners with disabilities, and (4) Effectiveness of classroom management.

The analysis of the collected data explores key themes related to the inclusion of learners with SCDs in educational settings. These themes, as indicated above, include the extent to which such learners are accommodated, the adequacy of teacher training and ongoing professional development, the availability and effectiveness of support systems, and the impact of classroom management strategies. Examining these aspects provides insight into both the challenges and best practices in fostering an inclusive learning environment. The following sections present a detailed analysis of each theme, highlighting patterns, relationships and emerging findings.

### Theme 1: Inclusion of learners with a social communication disorder

Teachers understood the importance of integrating learners with SCD into ordinary mainstream classrooms. Recognising the inclusion of learners with SCD was considered more beneficial than exclusion, as it could improve verbal and nonverbal communication. Additionally, teachers indicated that these learners’ difficulties with social interaction frequently interrupted classroom activities because they occasionally took turns in conversations. The quotations that follow exemplify the teachers’ opinions about the necessity of including learners with SCD in their mainstream classrooms to mediate these learners’ challenges:

‘Yeah, unfortunately, we cannot exclude them because of their constitutional rights. So, we must make sure these learners participate together with those that do not have challenges.’ (P-3, school A, male)

‘My perception of inclusion of learners with social communication disorder … I think it is manageable provided that the root of the communication disorder is known.’ (P-7, school 6, female)

‘Firstly, I believe that each and every learner…each and every person must get an education, okay? So, for me to exclude them, I feel like I am failing them.’ (P-11, school D, female)

Some teachers were overwhelmed and reported having good and bad experiences when working with learners who had SCD in their primary school classrooms. According to the findings of the focus group discussions and the in-depth interviews, teachers primarily reported negative experiences since they were unsure of how to handle learners who had difficulties with social interaction and communication skills. Teachers’ experiences in the classroom are reflected in the statements that follow:

‘My positive experience is that all learners, regardless of their disabilities, should be included in the Inter-Sen Phase although some still portray the challenges of ability to initiate or sustain interactions with peers and not following simple instructions.’ (P-6, school B, non-binary)

‘As an Inter-Sen teacher, the positive aspect of accommodating such learners it is that they feel a sense of belonging…that there is someone who is paying attention to them. The negative part is that the learner can be too emotionally dependent on the educator.’ (P-7, school C, female)

Empirical evidence from field observations suggests that a subset of teachers may possess insufficient knowledge or pedagogical competence to effectively implement inclusive strategies for learners exhibiting challenges associated with SCD. The envisaged inclusive strategies included, but were not limited to, peer-mediated interventions, usage of augmentative and alternative communication (AAC) tools, and integration of explicit instruction on conversational turn-taking, that is, training learners to know when to speak and when to listen during a conversation.

### Theme 2: Teacher training and continuous professional development

The study results have shown the importance of prioritising teacher training programmes and continuous professional development regarding the teaching and learning environment’s skills, knowledge and values that enable inclusive education. An atmosphere like this will help teachers manage diversity in the classroom, allowing them to work with learners with various learning disabilities. The researchers have noted the participants’ opinions that teachers’ ongoing professional development is a prerequisite:

‘I really feel helpless when supporting these learners in my classroom because sometimes, instead of answering questions that use figurative language, they become emotional and withdrawn. They cannot differentiate between jokes and meaningful conversations. We really need in-service training on how to address these kinds of issues.’ (P-8, school C, male)

‘I feel that I’m not effectively trained to teach those learners who have challenges related to social communication because sometimes those learners need people who can address their issues with knowledge and confidence.’ (P-4, school B, male)

‘Not necessarily that we are trained, not trained in that manner. So, as teachers, we learn a lot of things from the work. It is sometimes frustrating to try to accommodate a learner who finds it difficult to follow simple instructions and cannot share clear ideas in group discussions. One learner in my classroom has these kinds of challenges.’ (P-6, school B, non-binary)

### Theme 3: Support provisioning for learners with disabilities

The findings have revealed the need for support structures from the Department of Education to enhance effective mediation strategies for addressing SCD issues found among learners in inclusive primary classrooms. The verbal quotes below attest to what the participants have said:

‘To be honest, there is the SBST [*School-based Support Team*] committee, but it is not as effective as it should be. So, I can’t say the school does assist us personally. Teachers have to come up with strategies for dealing with learners with behavioural problems.’ (P-11, school D, female)

Teachers will also benefit greatly from having an efficient and well-functioning school-based support team when applying various strategies to mediate challenges related to SCD. In another school (School B), the interviews revealed that teachers were determined to offer academic support to learners exhibiting SCD and to collaborate with the external professional bodies if the need arises. The excerpts below are the opinions shared by the participants:

‘I took a long time to understand this problem of some learners interrupting others or failing to respond when spoken to, sometimes I thought of giving them a lash, just that we are no longer allowed to do so. Up to so far, I heard about one occupational therapist in town who can help us on how to deal with these learners who do not pay attention when I teach.’ (P-5, school B, female)

Data obtained from focus group discussions indicated that learners presenting with challenges characteristic of SCD were referred to departmental leadership and school-based support teams for support provision, but the delayed response and intervention increased teacher workload and emotional strain. Concurrently, the classroom observations in one school (School C) revealed the inability of the teacher (P-8), who found it challenging to come up with strategies to support a learner in his classroom after she failed to respond empathetically to her classmate.

### Theme 4: Effectiveness of classroom management

Teachers expressed their opinions that using good classroom management as an intervention technique can serve as one of the mediation strategies to address learners with SCD in inclusive primary school classrooms. According to some teachers, for P-2 and P-7, establishing guidelines and ensuring learners abide by them can be highly beneficial. Teachers expressed their feelings as reported verbatim by the following quotes:

‘Effective classroom management … I think it is the core that can help to alleviate the challenging learning disabilities. My understanding of effective classroom management is that as a teacher, you need to get to class on time because, in most cases, the classroom that is left idling gives the impression that the learners therein are abandoned and not supported.’ (P-2, school A, male)

‘Effective classroom management creates a safe and inclusive space where all learners can succeed. I believe that classroom rules must be followed so that all the learners, including those with learning disabilities such as this one of social communication disorders, can be managed and supported.’ (P-8, school C, male)

The focus group discussions yielded valuable insights regarding the prevalence of learners with SCD in some of the inclusive primary schools and generally suggested mediation strategies to address such NDDs. Overall, the focus group discussion suggested that learners with SCD in inclusive primary classrooms should benefit from structured routines, explicit instructions and visual supports to enhance comprehension. It was further suggested that teachers could demonstrate social interactions, show learners with SCD how to interact with their peers or incorporate role-playing. Two teacher-participants in a focus group at ‘School B’ shared their ideas that using social stories to guide conversations and social cues can benefit learners with SCD. In another focus group discussion at ‘School D’, the DHs shared that peer support through group activities can promote inclusion, while individualised interventions like speech therapy and tailored communication strategies may help learners with SCD express themselves. It was believed that a patient and supportive environment may enhance a feeling of being valued and included in learners with SCD, both academically and socially.

Based on their personal experiences and/or perceptions in the classroom, participants showed that they were aware of behavioural issues. In schools A, C and D, the furniture could not be arranged in the classroom to support teacher-learner interaction because of overcrowding. Learners who appeared to be having SCD could not be given proper and effective support because of overcrowding. Although the teachers in different schools tried to apply various mediation strategies to support learners with SCD, pedagogical and environmental barriers kept on interjecting. The researchers had observed the teaching and learning environments in various selected inclusive primary schools, and the general feeling was that SCD, being a NDD, needs professional intervention. Teachers needed further training or in-service training on developing proper mediation strategies to address issues related to NDD and SCD in their inclusive learning environments.

## Discussion

The research findings highlight the importance of recognising and accommodating learners with SCD in inclusive primary school classrooms. Education White Paper 6 (Department of Education 2001) emphasises that classroom teachers are the key resource in implementing inclusive education and should be equipped to meet the diverse learning needs of all learners, including those facing learning barriers. Participants emphasised that when learners with SCD receive appropriate support, such as individualised communication strategies, structured routines and peer-assisted learning, they are better able to engage in classroom activities and social interactions. A report by ‘The Incredible Years’ (2025) states that being in a diverse classroom enables learners with SCD to develop appropriate social behaviours and communication skills by observing and interacting with their peers. The study also revealed that teachers play a crucial role in fostering an inclusive environment by using clear instructions, visual supports and social skills training. Concurrently, Hamstead (2024) asserts that inclusive education involves fostering a classroom environment and implementing teaching strategies that embrace and support the diverse neurological profiles of learners. Overall, the findings suggest that inclusive practices that acknowledge and address the unique needs of learners with SCD lead to more positive learning experiences and social integration. It should therefore be mandatory for inclusive education policies and frameworks to strengthen school-based support teams with clear referral pathways and accountability mechanisms to support learners presenting SCD in inclusive primary classrooms.

The research findings highlight the necessity of continuous professional development for teachers to support learners with SCD effectively. Participants highlighted that ongoing training enhances teachers’ understanding of SCD and equips them with strategies to foster inclusive learning environments. The outcomes of the study conducted in the United States by Johnson, Williams and Wilson (2024) have revealed that inclusive education training programmes are designed to provide teachers with the expertise, strategies and mindset needed to effectively support and teach learners with diverse learning needs, including those, in the context of this study, with SCD. Key areas of focus include communication techniques, behaviour management and visual supports to aid interaction. The findings of this study also highlight the importance of continuous professional development, which enables teachers to provide tailored support, promoting the academic and social success of learners with SCD. Murphy (2024) confirms that teachers require continuous professional development to acquire new strategies and resources for supporting diverse learners, including differentiated instruction, assistive technologies and inclusive classroom management techniques. Participants stressed the need for specialised training programmes to equip teachers with strategies for managing SCD in classrooms. These approaches enable teachers to meet the unique needs of learners with disabilities, learning difficulties or language barriers, ensuring equitable access to education for all.

The research findings emphasise the importance of support provisioning for learners with SCD in educational settings. Murphy (2024) advocates that learners with disabilities often need individualised support to engage fully in the classroom, and teachers should prioritise equity by providing assistance tailored to each student’s specific needs. According to Vygotsky’s (1978) socio-cultural theory, teachers can create inclusive, scaffolded learning activities that build on learners’ strengths while subtly extending their social communication skills as they approach the ZPD. A learner with SCD could find it difficult to start or maintain a brief conversation, even though they might be able to independently welcome peers with individual words (such as ‘Hello’) (Li et al. 2023). With the help of a teacher or peer model, the learner can move beyond simple one-word greetings in the ZPD and participate in a quick, structured conversation (e.g. ‘Hi, how are you?’ → ‘I’m good, thank you’). Zhou, McCarthy and Durbin (2022) attest that with regular scaffolding, modelling and practice under supervision, the learner eventually internalises this communication ability and gains the capacity to conduct simple conversations on their own. According to Vygotsky (1978), learning occurs in socio-cultural contexts where learners engage in discourse and cooperation with more experienced people. The relationship between social interactions and cognitive development is emphasised by Vygotsky’s socio-cultural theory.

Participants highlighted the need for active involvement from interested parties within the Department of Education to address the challenges faced by these learners. Effective support requires collaboration among teachers, school-based support teams and policymakers to create inclusive environments tailored to the needs of learners with SCD. According to Yoro et al. (2020), support refers to any measures that enhance a school’s capacity to meet the diverse learning needs of learners. It also involves having a team of teachers who are prepared and willing to assist and accommodate learners facing learning barriers. Adequate resources, including speech therapists and communication aids, are essential for addressing the unique needs of learners with SCD. The Department of Education must prioritise developing and enforcing policies that support inclusive education practices. These findings emphasise the need for a multidimensional approach to ensure that learners with SCD receive the support they need to thrive academically and socially.

The research findings highlight the effectiveness of classroom management as a key intervention strategy for supporting learners with SCD in inclusive primary school classrooms. Smith, Thompson and Maynard (2022) reiterate that school self-management interventions can tackle behavioural issues by assisting learners in developing essential social, emotional and behavioural skills. They uphold that typically, school-based self-management programmes aimed at learners with challenging behaviours [SCD in the context of this study] yield positive effects on both behavioural and academic outcomes. Participants emphasised that structured routines, clear expectations and positive reinforcement help create an environment where these learners can thrive. Strategies such as visual schedules, consistent communication cues and designated quiet spaces were identified as beneficial in reducing anxiety and enhancing participation. In the same vein, Thomas and Karuppali (2022) suggest the use of a visual activity schedule (VAS) intervention programme as a commonly utilised approach for teaching various skills, including staying on task, following schedules, transitioning between activities, initiating social interactions, engaging in independent play, and developing classroom and academic skills. Moreover, encouraging peer support and implementing collaborative learning methods were viewed as effective ways to enhance social interaction. Overall, the study indicates that effective classroom management strategies are crucial mediation tools for academically and socially supporting learners with SCD.

### Strengths and limitations

The qualitative research methodologies employed in this study, utilising a case study research design, provided deep and contextually rich insights into effective remediation strategies for supporting learners with SCD in inclusive primary classrooms. Lim (2024) opined that qualitative methods have become essential for gaining profound insights and comprehending intricate phenomena. The case study research design allowed for integrating multiple data sources, such as in-depth interviews, focus group discussions and observations, ensuring a comprehensive analysis that enhanced the validity of the findings (Rashid et al. 2019). Despite the constructive views shared by the teachers regarding the need for inclusion of learners with SCD in primary school classrooms, the study’s findings have revealed the need for enhancement of support for learners with SCD. It is, however, worth noting that the relatively small sample size of the study presented limitations. The sample size preferred for this study may not accurately represent all primary school teachers in the Bojanala District of North West province of South Africa. It is, therefore, impossible to generalise the study’s findings. Future research should focus more on continuous professional training for teachers in mainstream schools in inclusive education. This should encompass the implementation of the Universal Design for Learning (UDL) in all primary school phases. The study establishes that learners with SCD have substantially better academic and social outcomes when inclusive tactics and strategies are tailored to their pragmatic and social communication needs.

### Recommendations

This study recommends:

- That inclusive policy and legislation should be taken into account in all endeavours to effectively implement mediation strategies for addressing SCD in inclusive primary classroom settings. When inclusive approaches are carefully used, peers start to perceive learners with SCD more favourably, which results in genuine friendships and less stigma.
- The need to prioritise teacher training programmes and ongoing professional development to enhance the skills, knowledge and values essential for fostering an inclusive teaching and learning environment. Teachers become more prepared to foster inclusive environments when they are trained to identify and address the invisible aspects of SCD, such as challenges with taking turns, maintaining focus or interpreting social cues. This emphasises the necessity of professional development that goes beyond inclusion on the surface. Institutions of higher learning should make it mandatory for teachers to complete training courses on recognising and assisting learners with SCD. Assure continuous capacity-building by providing access to interdisciplinary knowledge, mentorship and workshops.
- That future research on SCD should examine the role of multidisciplinary collaboration among teachers, therapists and psychologists in improving school or academic performance for learners with specific learning disabilities. Collaboration with external stakeholders, such as psychologists or neuropsychologists, may enhance support for learners with SCD by providing assessments, emotional support and interventions for co-occurring issues like anxiety, Attention-deficit Hyperactivity Disorder (ADHD) or autism spectrum traits.
- Considering and analysing the relationship between classroom management strategies and learner engagement in inclusive settings. Future comparative studies should focus on the effectiveness of traditional behaviour management techniques compared with newer, inclusive approaches. These include, but are not limited to, proactive and responsive strategies that foster a positive school climate, build relationships, and enhance collaboration, empathy and accountability to support all learners, including those with disabilities.

## Conclusion

The study has explored the effectiveness of mediation strategies for addressing SCD in inclusive primary school classrooms. Various strategies were suggested for future curbing of deficits related to support for learners with SCD, including recognition and implementation of inclusive policies and legislation. The study brings attention to the often-overlooked needs of learners with SCD, distinguishing their challenges from broader categories like autism or general speech-language impairments. According to this study, learners who exhibit social communication difficulties may suffer long-term effects on their ability to fulfil academic goals if teachers perceive them negatively in their classrooms. This contributes to the improvement of the conception and implementation of inclusion. It offers empirical proof that inclusive practices created especially for learners with SCD, like peer-mediated support and systematic social skills training, enhance learning outcomes and promote social integration. This reaffirms the importance of tailored strategies within inclusive frameworks. Future studies should look into the long-term emotional, social and academic results of learners with SCD who receive inclusive care. This could reveal whether early interventions lead to sustained improvements in peer relationships, self-esteem and academic achievement.
